# Inflammatory Activity on Natalizumab Predicts Short-Term but Not Long-Term Disability in Multiple Sclerosis

**DOI:** 10.1371/journal.pone.0169546

**Published:** 2017-01-12

**Authors:** Joel Raffel, Arie R. Gafson, Samer Dahdaleh, Omar Malik, Brynmor Jones, Richard Nicholas

**Affiliations:** Department of Medicine, Imperial College London, London, United Kingdom; Westmead Millennium Institute for Medical Research, AUSTRALIA

## Abstract

**Background:**

In people with multiple sclerosis treated with interferon-beta or glatiramer acetate, new MRI lesions and relapses during the first year of treatment predict a poor prognosis.

**Objective:**

To study this association in those receiving natalizumab.

**Methods:**

Data were collected on relapses, new MRI activity, and Modified Rio Score after initiation of natalizumab in an observational cohort of 161 patients with high baseline disability. These were correlated with Expanded Disability Status Scale (EDSS) progression at years 1, 2, 3, and 3–7 after treatment initiation, versus pre-treatment baseline.

**Results:**

46/161 patients had a relapse in the first year and 44/161 had EDSS progression by year 2. Relapses and Modified Rio Score in the first year of treatment predicted EDSS progression at year 1 and 2 after treatment initiation. However, this effect disappeared with longer follow-up. Paradoxically, there was a trend towards inflammatory activity on treatment (first year Modified Rio Score, relapses, and MRI activity) predicting a *lower* risk of EDSS progression by years 3–7, although this did not reach statistical significance. Those with and without EDSS progression did not differ in baseline age, EDSS, or pre-treatment relapse rate. Relapses in year 0–1 predicted further relapses in years 1–3.

**Conclusions:**

Breakthrough inflammatory activity after natalizumab treatment is predictive of short-term outcome measures of relapses or EDSS progression, but does not predict longer term EDSS progression, in this cohort with high baseline disability.

## Introduction

In recent years, a number of new treatments have emerged for patients with relapsing multiple sclerosis (RMS).[1] Their development was underpinned by targeting MRI activity in phase 2 studies, leading to phase 3 studies which demonstrated reductions in relapse frequency and a variable effect on time to disability progression. The principal argument for their long term use is that treatments that target inflammatory activity probably improve long term disability outcomes, at least at a population level.[2, 3]

These principles have been extended in the pursuit of personalised medicine in multiple sclerosis (MS), where it is hypothesised that on-treatment breakthrough inflammatory activity can be used to predict poor long-term disability outcomes at an individual level.[4] This is supported by observational studies on therapies interferon-beta and glatiramer acetate, where early on-treatment relapses, MRI activity, and Kurtzke Expanded Disability Status Scale (EDSS) disability progression have been shown to predict poor medium-term clinical outcomes of relapses and/or EDSS progression in individuals.[5–11] For example, Rio and colleagues showed that combined scores of MRI lesions, relapses, and/or EDSS progression after interferon-beta initiation predicted further EDSS progression at two years.[9] Similarly, combined scores of MRI lesions and relapses after glatiramer acetate predict “clinical activity” (defined as relapses or EDSS progression) after 2 years.[6] Most of these studies are limited in that their follow-up periods were between 2–3 years.[6–11] Their applicability to other therapies such as teriflunomide, dimethyl fumarate, fingolimod, natalizumab, and alemtuzumab is also uncertain. If on-treatment breakthrough inflammatory activity does predict poor long-term disability outcomes, the next step would be to evaluate whether switching treatments can improve long-term prognosis, as proposed by the no evidence of disease activity (NEDA) approach.[12] However, for the treating physician, it is currently unclear whether on-treatment inflammatory breakthrough activity should trigger a change in medication, or not.

Natalizumab is a highly active therapy that is widely used in patients with RMS. It is effective in reducing relapses, MRI activity, and time to EDSS progression with a higher efficacy than interferon-beta.[13, 14] The objective of this observational study was to evaluate whether early relapses or MRI activity after starting natalizumab treatment predicts EDSS progression at later time points, in a cohort with high baseline disability.

## Patients and Methods

Data were collected from an observational cohort of 204 patients initiating natalizumab between March 2007 and October 2010 at Imperial College Healthcare NHS Trust, with up to 7 years of follow-up. Data on relapse rate and EDSS were prospectively documented at routine 6-monthly clinic visits. EDSS data were collected until December 2014. MRI was routinely performed before treatment and at one year. Data were retrospectively collated for the purpose of this study. Patients were excluded if they had less than three years follow-up, or were treated with natalizumab for less than one year.

Data were analysed as part of a clinical audit, registered and ethically approved at Imperial College Healthcare NHS Trust, for which written informed consent was not required (Audit registration number 1987–2015). Anonymised clinical data are not available on a public repository since ethical approval was not granted by Imperial College Healthcare NHS Trust Research Office to share individual patient disease characteristics outside of their healthcare institution, since these may contain identifying or sensitive patient information. Requests for data may be sent to richard.nicholas@imperial.nhs.uk.

### Early markers of inflammatory disease activity

Relapses were defined as an acute worsening of function lasting at least 48 hours, in the absence of fever or infection. MRI activity was defined as the presence of 1 active lesion (either new or enlarging T2 lesions) relative to a baseline MRI scan. A combined score of MRI and relapse activity was also applied to this first year after natalizumab therapy (Modified Rio Score–Table 1), which employs more stringent criteria to define new MRI activity.[7]

**Table 1:** Modified Rio Score[7].

**Modified Rio Score scoring criteria**		
**Criterion**	Change over 1^st^ year	Score
**MRI**	≤ 4 new T2 lesions	0
	> 4 new T2 lesions	1
**Relapse**	No relapses	0
	1 relapse	1
	≥ 2 relapses	2
**Score = MRI criterion + relapse criterion**		

### Disease progression

Disability progression was defined as an increase of 1 EDSS point in those with EDSS <5.5, or an increase of 0.5 EDSS point in those with EDSS ≥5.5. EDSS progression was confirmed over 6 months at repeat clinic visits. Patients were labelled as disability progression responders and non-responders after 1 year of treatment (Year 1 EDSS Progression) by comparing EDSS captured after 1 year with pre-natalizumab EDSS. This was repeated after 2 years, 3 years, and 3–7 years, by comparing the latest available EDSS rating (up to 7 years after treatment initiation) with pre-natalizumab EDSS.

### Statistical analysis

Demographic data are presented as mean +/- standard deviation (SD). Difference between means was assessed using unpaired Student’s t-test, after testing for normality of the data. To investigate the association between early markers of disease activity and disability progression data were analysed in Kaplan-Meier curves using Log-Rank test and Cox regression, and also in 2x2 contingency tables using Fisher’s exact test with odds ratios (OR) and 95% confidence intervals (CI) calculated. Chi-squared test for trend was used for 3x2 contingency tables of the Modified Rio Score and for analysis of change in categorical data over time. Corrections for multiple comparisons were not made, since comparisons were complementary and a consistency of results was apparent between different groups.[15, 16] For the purpose of displaying results in graphical format, results were converted to percentages. A logical regression multivariable model was also used to investigate for multivariate associations that predict disability outcomes. SPSS and R statistical package were used for these analyses.

## Results

### Demographics

**Fig 1:**
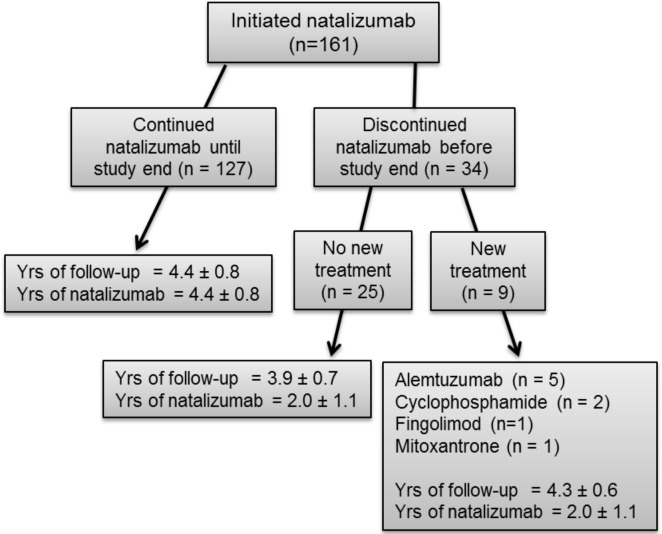
161 participants were included in all analyses. The treatment pathway, and mean follow-up time, is detailed in the figure.

During the study period, 204 patients started natalizumab treatment. Subjects were excluded from analysis if they had discontinued natalizumab within 1 year (n = 16), if there were less than 3 years of clinical follow-up data (n = 20), or if there were insufficient clinical or radiological data (n = 7), leaving 161 patients for analysis. Of these, 127 received natalizumab throughout the study period, 9 stopped natalizumab after 1+ year of treatment and switched to a new disease modifying medication, and 25 stopped natalizumab after 1+ year of treatment but did not receive new disease modifying medication (Fig 1). No differences existed in demographic, clinical and MRI data between those included in and excluded from the study. There were no differences between Year 3–7 EDSS Progression Responder and Year 3–7 EDSS Progression Non-Responder cohorts, other than an expected difference in most recent EDSS (Table 2). Logistic regression multivariable analysis that included age, sex, disease duration, number of previous treatments, pre-natalizumab relapse rate, and pre-natalizumab EDSS confirmed that none of these baseline measures predicted response to treatment.

**Table 2:** Characteristics of study cohort, presented as mean ± SD. Percentages are rounded to the nearest integer, and may not add to 100%.

	**Full Cohort**	**Year 3–7 EDSS Progression Responders**	**Year 3–7 EDSS Progression Non-Responders**
**Sample Number**	161	101	60
**Mean Age (Years)**	40.6 ± 10.2	39.7 ± 10.8	42.1 ± 9.1
**Sex**			
**Female**	101 (63%)	67 (66%)	34 (57%)
**Male**	60 (37%)	34 (34%)	26 (43%)
**Disease Duration (years)**	8.9 ± 6.1	8.7 ± 6.0	9.4 ± 6.2
**Number of previous treatments**			
**0**	42 (26%)	30 (30%)	12 (20%)
**1**	89 (55%)	52 (51%)	37 (62%)
**2**	21 (13%)	14 (14%)	7 (12%)
**3**	8 (5%)	4 (4%)	4 (7%)
**4**	1 (1%)	1 (1%)	0
**Relapses 2 years prior to natalizumab**	3.0 ± 1.3	3.0 ± 1.3	2.9 ± 1.2
**Pre natalizumab EDSS**	4.2 ± 1.8	4.1 ± 1.9	4.4 ± 1.6
**Most recent EDSS**	4.5 ± 2.0	3.7 ± 2.0	5.8 ± 1.4 ****
**Years of follow-up after natalizumab**	4.3 ± 0.8	4.3 ± 0.8	4.3 ± 0.9

When divided into Year 3–7 EDSS Progression Responders and Non-Responders, there were no significant differences between characteristics, other than ‘most recent EDSS’ (**** = p<0.0001).

### Markers of inflammatory disease do not affect disability progression survival analysis

**Fig 2:**
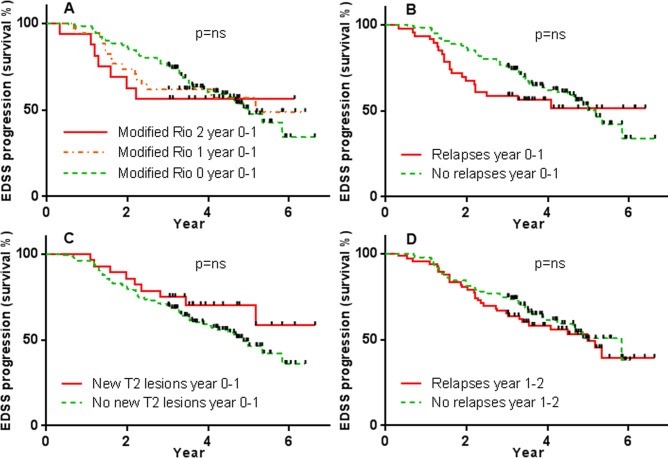
On-treatment inflammatory disease activity does not affect overall disability survival analysis. First year after natalizumab initiation Modified Rio Score (A), relapses (B), and MRI activity (C), and second year after natalizumab initiation relapses (D), versus survival from EDSS progression over time. Black markers on survival lines represent the duration of follow-up data for those participants who have not undergone EDSS progression (i.e. the time of subject censorship in survival analysis).

46 of 161 patients had a relapse in the first year, and 28 of 161 had new MRI activity. Modified Rio score was 1 in 34 patients, and 2 in 16 patients. Markers of inflammatory disease in the first year (Modified Rio Score, relapses, MRI activity) and second year (relapses) had no significant effect on disability progression plotted as a survival analysis (Mod Rio Score year 0–1: risk ratio (RR) 1.15, log-rank p = 0.74, Cox regression p = 0.44; Relapses year 0–1: RR 1.3, log-rank p = 0.31, Cox regression p = 0.31; MRI activity year 0–1: RR 0.64, log-rank p = 0.21, Cox regression p = 0.24; Relapses year 1–2: RR 1.17, log-rank p = 0.51, Cox regression p = 0.51; Fig 2A–2D). Those with low Modified Rio Scores, and without relapses in year 0–1, appeared to have lower risk of disability progression in the first two years, but this effect disappeared with longer follow-up (Fig 2A and 2B).

### Modified Rio Score predicts short-term but not medium-term disability progression

A limitation of Kaplan-Meier curves is that events (in this case disability progression) are treated as irreversible, which is inappropriate given that EDSS can improve with treatment. In addition, it was observed that curves converge and cross in both the Modified Rio Score and relapses survival analyses (Fig 2A and 2B), suggesting that any association between inflammatory biomarkers and disability progression may change over time. Therefore, contingency tables were used to investigate the relationship between inflammatory biomarkers and EDSS progression at specific time points of years 1, 2, 3, and 3–7.

**Fig 3:**
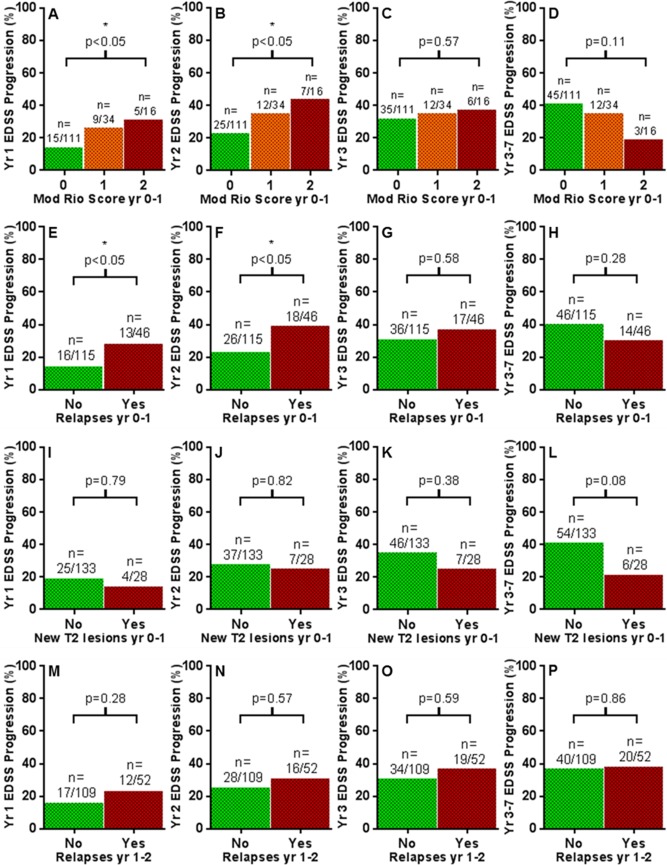
On-treatment inflammatory disease activity predicts short-term but not long-term disability outcomes. Early measures of on-treatment inflammatory disease in first year (A-L) and second year (M-P) after treatment initiation, versus percentage of future EDSS Progression in year 1 (A, E, I, M), year 2 (B, F, J, N), year 3 (C, G, K, O), and years 3–7 (D, H, L, P). * denotes p<0.05.

Modified Rio Score in the first year of treatment predicted EDSS progression at year 1 and 2 (Year 1: 15/111 vs 9/34 vs 5/16 EDSS progression for those with Mod Rio Score of 0, 1, 2 respectively. Mod Rio Score 1, Odds Ratio (OR) 2.3, 95% Confidence Interval (CI) 0.9–5.9; Mod Rio Score 2, OR 2.9, CI 0.9–9.6; p<0.05. Year 2: 25/111 vs 12/34 vs 7/16 EDSS progression for those with Mod Rio Score of 0, 1, 2 respectively. Mod Rio Score 1, OR 1.9, CI 0.8–4.3; Mod Rio Score 2, OR 2.7, CI 0.9–7.9; p<0.05; Fig 3A and 3B). However, it did not predict EDSS progression at year 3, or year 3–7 (Year 3: 35/111 vs 12/34 vs 6/16 EDSS progression for those with Mod Rio Score of 0, 1, 2 respectively. Mod Rio Score 1, OR 1.2, CI 0.4–2.7; Mod Rio Score 2, OR 1.3, CI 0.4–3.9; p = 0.57; Year 3–7: 45/111 vs 12/34 vs 3/16 EDSS progression for those with Mod Rio Score of 0, 1, 2 respectively. Mod Rio Score 1, OR 0.8, CI 0.4–1.8; Mod Rio Score 2, OR 0.3, CI 0.1–1.3; p = 0.11; Fig 3C and 3D). If anything, there was a paradoxical trend towards *lower* Modified Rio Score predicting EDSS progression in years 3–7, although this was not statistically significant (Fig 3D). This shift over time in the polarity of the predictive value of Modified Rio Score on disability was caused by year-on-year increases in the proportion of non-responders within the Mod Rio Score 0 group (p<0.0001), versus no significant year-on-year change in the proportion of non-responders within the Mod Rio Score 1–2 group (p = 0.90).

### Relapses and MRI activity predict short-term but not medium-term disability progression

Similar trends were observed when studying on-treatment relapses and MRI activity in isolation. Relapses in the first year of treatment predicted EDSS progression at year 1 and 2 (Year 1: 16/115 vs 13/48 EDSS progression for those without and with relapses respectively. OR 2.4, CI 1.1–5.6, p<0.05. Year 2: 26/115 vs 18/48 EDSS progression for those without and with relapses respectively. OR 2.2, CI 1.1–4.6, p<0.05; Fig 3E and 3F). However, they did not predict EDSS progression at year 3, or years 3–7 (Year 3: 36/115 vs 17/48 EDSS progression for those without and with relapses respectively. OR 1.3, CI 0.6–2.6, p = 0.58. Year 3–7: 46/115 vs 14/48 EDSS progression for those without and with relapses respectively. OR 0.7, CI 0.3–1.4, p = 0.28; Fig 3G and 3H). If anything, there was a paradoxical trend towards *lack* of relapses predicting EDSS progression at years 3–7, although this was not statistically significant (Fig 3H). New MRI activity in the first year of treatment did not predict EDSS progression at any future time point (Year 1: 25/113 vs 4/28 EDSS progression for those without and with new MRI activity respectively. OR 0.7, CI 0.2–2.3, p = 0.78 Year 2: 37/113 vs 7/28 EDSS progression for those without and with new MRI activity respectively. OR 0.86, CI 0.3–2.2, p = 0.82. Year 3: 46/113 vs 7/28 EDSS progression for those without and with new MRI activity respectively. OR 0.6, CI 0.2–1.6, p = 0.38. Year 3–7: 54/113 vs 6/28 EDSS progression for those without and with new MRI activity respectively. OR 0.4, CI 0.2–1.0, p = 0.08; Fig 3I–3L). Again, there was a paradoxical trend towards *lack* of new MRI activity predicting EDSS progression at years 3–7, although this was not statistically significant (Fig 3L). As before, this shift over time in the polarity of the predictive value of relapses and MRI activity on disability was caused by year-on-year increases in the proportion of non-responders within the ‘no relapses group’ (p<0.0001) and ‘no new MRI activity group’ (p<0.0001), versus no significant year-on-year change in the proportion of non-responders within the ‘relapses group’ (p = 0.89) and the ‘new MRI activity group’ (p = 0.54).

Similarly, the subgroup of patients with new gadolinium-enhancing MRI lesions in the first year of treatment (n = 14) had no difference in EDSS progression in future years (Year 1: 25/147 vs 4/14 EDSS progression for those without and with new gadolinium enhancing lesions respectively. OR 2.0, CI 0.6–6.7, p = 0.28. Year 2: 40/147 vs 4/14 EDSS progression for those without and with new gadolinium enhancing lesions respectively. OR 1.1, CI 0.3–3.6, p = 1.0. Year 3: 48/147 vs 4/14 EDSS progression for those without and with new gadolinium enhancing lesions respectively. OR 0.8, CI 0.2–2.8, p = 1.0.Year 3–7: 56/147 vs 4/14 EDSS progression for those without and with new gadolinium enhancing lesions respectively. OR 0.65, CI 0.2–2.2, p = 0.57). Relapses in the second year of treatment were also unable to predict EDSS progress at future time points (Year 1: 17/109 vs 12/52 EDSS progression for those without and with relapses respectively. OR 1.6, CI 0.7–3.7, p = 0.28. Year 2: 28/109 vs 16/52 EDSS progression for those without and with relapses respectively. OR 1.3, CI 0.6–2.7, p = 0.57. Year 3: 34/109 vs 19/52 EDSS progression for those without and with relapses respectively. OR 1.3, CI 0.6–2.5, p = 0.59. Year 3–7: 40/109 vs 20/52 EDSS progression for those without and with relapses respectively. OR 1.1, CI 0.5–2.1, p = 0.86; Fig 3M–3P).

### Results are not affected by change in medication, baseline disability, or neutralising antibodies

**Fig 4:**
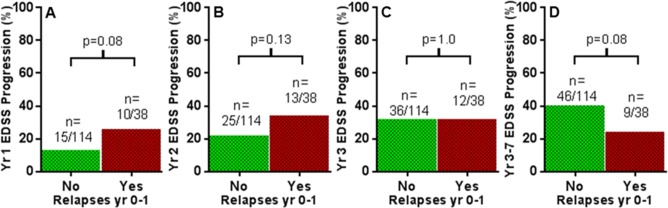
Results remain consistent after exclusion of those that switched to other treatment. After exclusion of patients who switched from natalizumab to other disease modifying treatment. On-treatment relapses in first year after treatment initiation versus percentage of future EDSS Progression in year 1 (A), year 2 (B), year 3 (C), and years 3–7 (D).

As may be expected, patients with inflammatory activity on natalizumab treatment were more likely to switch to other disease modifying drugs, such as alemtuzumab, fingolimod, or cyclophosphamide. For example, 8/46 of those with relapses in year 0–1 switched to an alternative treatment at some point during the 3–7 year follow-up period of this study, versus 1/115 of those with no relapses. However, the trends observed in this study remained consistent with subgroup analyses after exclusion of those that switched to other medications (for example as represented in Fig 4). Trends also remained consistent with subgroup analysis restricted to those with baseline EDSS scores <4 (n = 68) or ≥4 (n = 93), and also after exclusion of those with neutralising antibodies (n = 6). There was no significant difference in mean follow-up time between those with different Modified Rio Scores, or indeed any of the other individual inflammatory measures.

Logistic regression multivariable model considered variables relating to demographics, disease history, and markers of inflammatory disease (age at natalizumab initiation, sex, pre-natalizumab relapse rate, pre-natalizumab MRI lesion count, pre-natalizumab MRI enhancing lesion count, number of previous treatments, post-natalizumab relapse rate, and post-natalizumab new MRI lesions), and found no further predictive relationships for disability outcomes (multiple r-squared: 0.051, p = 0.46).

### Relapses in the first year of treatment with natalizumab predict further relapses

Relapses in year 0–1 of treatment were correlated with the risk of further relapses in years 1–3. 35 of 115 (30%) with no relapses in year 0–1 reported relapses in years 1–3. In contrast, 31 out of 46 (67%) with relapses in year 0–1 reported relapses in years 1–3 (OR 4.7, CI 2.3–9.8, p<0.0001).

## Discussion

This is the first study to report on-treatment predictive measures of long term disability outcomes in a cohort of patients on natalizumab. We find that relapses and Modified Rio Score in the first year of natalizumab treatment predict year 1 and year 2 EDSS progression. However, this effect disappears after three years of follow-up. If anything, there is a consistent paradoxical trend towards on-treatment relapses, MRI activity, and high Modified Rio Score predicting *better* 3–7 year disability outcomes, versus pre-treatment baseline EDSS, although this did not reach statistical significance. This shift over time was driven by highly significant year-on-year increases in disability progression specifically in those *without* on-treatment inflammatory activity. In this group it was less likely for the EDSS to worsen in the first 1–2 years, but far more likely for the EDSS to worsen over each subsequent year. Results were consistent when restricted to those who remained on natalizumab throughout the follow-up period. Given this, our data suggests a disconnect between natalizumab’s ability to suppress focal inflammatory activity underlying relapses and new T2 MRI lesions, and its putative effect on the progression of long-term disability.

Previous studies looking at predictive measures of treatment response have tended to focus on earlier generation MS treatments, namely interferons and glatiramer acetate. Like ours, these studies identified on-treatment MRI activity and relapses as medium-term (≤3 years) prognostic markers of EDSS progression and/or further relapses.[5–11] However, the majority had limited follow up and thus conclusions on long term disability could not be made.[6–11] It is possible that the predictive effect of relapses and MRI activity on short-term EDSS outcomes could reflect a predictive effect partly on relapse-mediated worsening of disability, rather than true progressive neurodegeneration. Related to this is a potential reporting bias, especially in studies based in the working neurology clinic, where it can be challenging to distinguish relapses from a true worsening of EDSS, and so both may be reported interdependently. This reporting bias decreases with long-term follow-up. In addition, we found that relapses in year 0–1 predict further relapses in years 1–3, in common with several of the above studies.[6, 7, 9, 11] Again, a patient reporting bias is likely to contribute to this association, and long-term disability progression should remain the preferred primary outcome. It would be of interest whether long-term follow-up data from the above studies would find results consistent with ours. Equally, it could be that the prognostic importance of relapses and MRI activity differs between interferon, glatiramer acetate, and natalizumab treatment.

Year 0–1 on-treatment MRI activity did not predict poor disability outcomes at any time point. This expands upon a previous post hoc analysis of randomized controlled trial data showing the clinical efficacy of natalizumab on year 0–2 relapses even in those with on-treatment MRI activity.[17] Of course, some new T2 lesions might have developed before natalizumab became effective, and future datasets should aim to “re-baseline” the patients with MRI scans performed 6 months after natalizumab initiation.[18] However, since gadolinium enhancing MRI also did not predict poor disability outcomes in our study, this suggests the lack of correlation between new MRI activity and long-term prognosis is real. Similarly, some relapses in years 0–1 may have developed before natalizumab became effective, but the data on year 1–2 relapses corroborates the finding of lack of predictive value of relapses on long-term disability.

Several caveats exist in this study, and must be considered. The cohort size is modest, and results should be replicated in larger cohorts. Our cohort had a high baseline EDSS (mean baseline EDSS 4.2) in comparison to interferon and glatiramer acetate studies (mean baseline EDSS 2 to 3), reflecting the fact that at the time of data collection, there were limited treatment options available for highly active relapsing-remitting and relapsing-progressive MS. Our cohort had poorer outcomes—46 of 161 patients had a relapse in the first year and 44/161 had EDSS progression by year 2—than previously described natalizumab cohorts with less baseline disability such as those in the pivotal randomised controlled trials.[13,14] Although some patients gained clear benefit from treatment it is likely that some patients within our cohort had a progressive component to their disease which was not responsive to treatment.[19, 20] Within this cohort, it could be that on-treatment breakthrough inflammatory activity is not a poor prognostic marker for long-term disability since it signifies that the patient does have an ongoing treatable focal inflammatory disease component, as opposed to others in the cohort who may have entered a predominantly irreversible neurodegenerative progressive stage of disease. That said, those with on-treatment inflammatory activity had equivalent baseline age and EDSS to those without on-treatment inflammatory activity in our cohort. In addition, results remained consistent when restricted to those with baseline EDSS <4. Another caveat arises in that follow-up time was variable, although always greater than 3 years. Kaplan-Meier curves tended to converge after this 3-year time point, bringing about the possibility that associations were affected by selection bias related to duration of follow-up, although this seems unlikely since follow-up duration was consistent between groups.

The observational nature of this study means we cannot speculate whether patients might have gained greater benefit from other treatments. Randomised controlled interventional studies are required to investigate whether those with breakthrough inflammatory activity would benefit from switching to other highly active treatments. Nevertheless, the data from this study argues that neither decrease in relapse rate, MRI activity, nor short-term stability of EDSS can be assumed to equate to better long-term prognosis. This has far-reaching implications, from the treating physician assessing those with on-treatment clinical activity, to the design and interpretation of the clinical trial.
